# Synaptotagmin-7 Counteracts Short-Term Depression during Phasic Dopamine Release

**DOI:** 10.1523/ENEURO.0501-23.2024

**Published:** 2024-03-06

**Authors:** Joseph J. Lebowitz, Sarah A. Kissiwaa, Kim A. Engeln, Anna M. Bowman, John T. Williams, Skyler L. Jackman

**Affiliations:** Vollum Institute, Oregon Health & Science University, Portland, Oregon 97239-3098

**Keywords:** dopamine, short-term plasticity, synaptotagmin

## Abstract

Dopamine neurons switch from tonic pacemaker activity to high-frequency bursts in response to salient stimuli. These bursts lead to superlinear increases in dopamine release, and the degree of this increase is highly dependent on firing frequency. The superlinearity and frequency dependence of dopamine release implicate short-term plasticity processes. The presynaptic Ca^2+^-sensor synaptotagmin-7 (SYT7) has suitable properties to mediate such short-term plasticity and has been implicated in regulating dopamine release from somatodendritic compartments. Here, we use a genetically encoded dopamine sensor and whole-cell electrophysiology in *Syt7* KO mice to determine how SYT7 contributes to both axonal and somatodendritic dopamine release. We find that SYT7 mediates a hidden component of facilitation of release from dopamine terminals that can be unmasked by lowering initial release probability or by predepressing synapses with low-frequency stimulation. Depletion of SYT7 increased short-term depression and reduced release during stimulations that mimic in vivo firing. Recordings of D2-mediated inhibitory postsynaptic currents in the substantia nigra pars compacta (SNc) confirmed a similar role for SYT7 in somatodendritic release. Our results indicate that SYT7 drives short-term facilitation of dopamine release, which may explain the frequency dependence of dopamine signaling seen in vivo.

## Significance Statement

Each midbrain dopamine neuron releases onto thousands of downstream cells, allowing the activity of dopamine neurons to exert outsized impacts on movement, motivation, and learning. Dopamine release scales nonlinearly with firing rates, suggesting these neurons might employ classical mechanisms of activity-dependent plasticity. Here, we show that dopamine release sites in multiple brain regions employ a well-characterized mechanism for plasticity, synaptotagmin-7, to dramatically boost dopamine release during high-frequency activity. This work generalizes a mechanism of short-term plasticity that has been well-characterized at conventional synapses to the release of neuromodulators and helps to explain the activity dependence of dopamine release.

## Introduction

Dopamine neurons fire tonically at low frequencies (∼1–8 Hz) and fire phasic bursts (>20 Hz) in response to salient stimuli (Fiorillo et al., 2003, 2008; Otomo et al., 2020). Phasic firing releases dopamine more effectively than tonic firing—stimulating dopaminergic axons with high-frequency bursts increases dopamine concentrations in terminal regions more than the same number of stimuli at low frequency (Gonon, 1988). Furthermore, elevated striatal dopamine levels driven by phasic stimuli drive conditioned place preference, while tonic stimulation does not (Tsai et al., 2009). These observations suggest that activity-dependent short-term plasticity, intrinsic to dopamine cells, may increase dopamine release during phasic firing.

At most presynaptic terminals, including those of dopamine neurons, action potential-induced Ca^2+^ influx triggers fast synchronous fusion by activating the low-affinity vesicular Ca^2+^ sensor synaptotagmin-1 (SYT1; Banerjee et al., 2020; Hikima et al., 2022; Liu et al., 2022; Delignat-Lavaud et al., 2023; Lebowitz et al., 2023). After Ca^2+^ influx, submicromolar “residual” Ca^2+^ persists in the presynaptic cytoplasm for hundreds of milliseconds and activates the high-affinity Ca^2+^ sensor synaptotagmin-7 (SYT7). SYT7 is inefficient at driving synchronous vesicle fusion on its own but can potentiate SYT1-triggered fusion if another action potential arrives within tens to hundreds of milliseconds (Jackman et al., 2016; Chen et al., 2017). This process, known as facilitation, is a nearly ubiquitous form of short-term plasticity that increases transmitter release during high-frequency activity (Jackman and Regehr, 2017). SYT7 has been shown to mediate facilitation at terminals that release glutamate (Jackman et al., 2016), GABA (Chen et al., 2017; Turecek et al., 2017) and acetylcholine (Caballero-Florán et al., 2023), but it remains unclear whether SYT7 performs a similar function in dopamine terminals.

*Syt7* RNA is expressed in many cell types in the brain but is enriched in SNc neurons (Saunders et al., 2018). Studies using *Syt7* KO animals or antibody-mediated inhibition suggest that SYT7 regulates dopamine release from the somatodendritic compartment (Delignat-Lavaud et al., 2022; Hikima et al., 2022). Further, high-frequency stimulation restores somatodendritic dopamine release in SYT1 KO animals, and high-frequency stimulation and DAT blockade reveal a component of release from terminals in SYT1 KO animals that is postulated to be under the control of SYT7 (Banerjee et al., 2020; Lebowitz et al., 2023). However, direct observation of SYT7-mediated short-term plasticity from dopamine terminals is lacking.

We used high-speed photometry of a fluorescent dopamine sensor in terminals, and whole-cell electrophysiology in the somatodendritic compartment of *Syt7* KO mice to probe the role of SYT7 in dopamine release evoked by low- and high-frequency stimuli. Release evoked by high-frequency stimulation was reduced in *Syt7* KO animals in both axon terminals and somatodendritic compartments. Paradigms that lowered the initial probability of release revealed facilitation of release that was absent in *Syt7* KO animals, and SYT7 enhanced dopamine release during transitions from tonic to phasic firing. Our results establish an important role for SYT7-driven plasticity in phasic dopamine release, namely, a hidden component of facilitation that limits the depression of release during phasic bursts.

## Materials and Methods

### Animals

Animals were group housed in a temperature-controlled room (24 ± 1°C) on a reverse light/dark cycle (lights on at 12:00 P.M.). *Syt7* KO mice (Chakrabarti et al., 2003) on a C57BL/6 background and WT littermates of either sex were used. Statistical tests were not used to predetermine sample size, but sample sizes were similar to those in previous studies (Ford et al., 2010; Patriarchi et al., 2018). Blinding and randomization were not performed unless otherwise indicated. All mice were handled in accordance with protocols approved by the Institutional Animal Care and Use Committee of Oregon Health and Science University.

### Stereotaxic injections

Adult mice (P30–P90) were anesthetized with ketamine/xylazine (100/10 mg/kg), placed in a stereotaxic apparatus (Kopf), and supplemented with 1–2% isoflurane. The scalp was shaved and disinfected with betadine and alcohol, an incision was made to expose the skull, and a small hole was drilled. 500 nl of AAV9.syn.dLight1.3b (4.66 10^13^ gc/ml; Patriarchi et al., 2018) was injected unilaterally using glass pipettes (Drummond Scientific) at a rate of 100 nl/min (Nanoject 3000, Drummond Scientific). Injection coordinates from bregma were +1.4 mm anterior, 1.4–1.5 mm lateral, and 3.5 mm ventral. Ten minutes after injection, the pipette was slowly retracted, and the scalp incision was closed with a gluture. Postsurgical analgesic (carprofen, 5 mg/kg) was administered subcutaneously for 48 h.

### dLight imaging

Acute brain slices were prepared from P60 to 120 mice 1–6 weeks after virus injection. Animals were anesthetized with isoflurane and euthanized. Brains were quickly removed into an ice-cold cutting solution containing (in mM) 125 choline Cl, 25 NaHCO_3_, 10 glucose, 2.5 KCl, 1.25 NaH_2_PO_4_, 2 Na pyruvate, 3 (3)-myo-inositol, 4.4 ascorbic acid, and 7 MgCl_2_, and 0.5 CaCl_2_, bubbled continuously with 95% O_2_/5% CO_2_. 270-µm-thick sagittal slices were prepared using a Leica VT1200S vibratome and transferred to a holding chamber with ACSF containing (in mM) 125 NaCl, 25 NaHCO_3_, 10 glucose, 2.5 KCl, 1.25 NaH_2_PO_4_, 2 Na-pyruvate, 3 (3)-myo-inositol, 4.4 ascorbic acid, 1 MgCl_2_, and 2 CaCl_2_, bubbled continuously with 95% O_2_/5% CO_2_. For low-Ca^2+^ experiments, ACSF was the same but contained (in mM) 2.5 MgCl_2_ and 0.5 CaCl_2_. Slices were stored at room temperature (∼24°C) prior to experiments.

Slices were transferred to a recording chamber and superfused with oxygenated ACSF (∼2 ml/min, 34 ± 1°C) containing 200 µM hexamethonium, 2 µM CGP, and 0.2 µM sulpiride. A bipolar stimulating electrode was placed in the dorsal striatum, and axons were stimulated using a constant-voltage stimulus isolator (Digitimer DS2A). dLight fluorescence was acquired through a 60× objective on an Olympus BX51WI microscope, equipped with a 488 nm LED (Thorlabs) and a FITC XF100-2 filter set (Omega). Fluorescent signals were acquired at 10 kHz using a custom-built amplified photodiode detector, digitized with an ITC-16 (InstruTech), and recorded in Igor Pro 8 (WaveMetrics) using custom routines (mafPC, courtesy of Matthew Xu-Friedman). Each interstimulus interval was repeated for multiple trials (typically 4–10 per experiment). Paired-pulse stimulations were presented in pseudorandom order at 20 s intervals. Phasic train stimulations were presented at 1 min intervals. Traces from individual trials were averaged to reduce recording noise and variability, though we observed little variability in response amplitudes and paired-pulse ratios across trials. Paired-pulse ratios did not depend on response amplitudes as determined by linear regression analysis (WT, *R*^2^ = 0.06; Syt7 KO, *R*^2^ = 0.16). Recordings were analyzed in Igor Pro. Signals were filtered using a 100 Hz low-pass filter, and photobleaching within trials was compensated by subtracting double-exponential curves fit to baseline fluorescence signals.

### SNc whole-cell recordings

Acute SNc slices were prepared from P30 to 70 mice as previously described (Robinson et al., 2019; Lebowitz et al., 2023). Animals were anesthetized with isoflurane before rapid decapitation into warmed (∼35°C) Krebs buffer containing (in mM) 126 NaCl, 1.2 MgCl_2_, 2.4 CaCl_2_, 1.4 NaH_2_PO_4_, 25 NaHCO_3_, and 11 dextrose. The brain was extracted and 222-µm-thick slices were cut in warmed Krebs in the horizontal plane with continuous bubbling of 95% O_2_/5% CO_2_. Slices were allowed to recover for at least 30 min at 33°C in bubbled Krebs. Typical preparations yielded three slices, which were hemisected for recording experiments. Brain extraction and slicing were all performed in the presence of 10 µM MK-801 to protect from NMDA-mediated excitotoxity. Recordings were obtained from hemisected slices continuously perfused with Krebs buffer at 34°C containing 10 µM CNQX, 100 µM picrotoxin, and 300 nM CGP55845 to block AMPA-R, GABA_A_-R, and GABA_B_-R, respectively. The internal solution used for recording contained (in mM) 100 K-methanesulfonate, 20 NaCl, 1.5 MgCl_2_, 10 HEPES (K), 10 BAPTA, 2 ATP, 0.2 GTP, and 10 phosphocreatine. GΩ seals were obtained using glass electrodes (initial resistance 1.1–1.4 MOhm) and allowed to stabilize for 1–2 min before break-in. No overt difference in cell firing behavior was observed between genotypes in cell-attached configuration (data not shown). Series resistance, capacitance, and membrane resistance were measured shortly after break-in using the average of 20 5 mV test pulses and were monitored during and upon completion of all recordings. Cells were voltage-clamped at −55 mV using an AxoPatch 1D amplifier, and the current was recorded at 1 kHz. DA cell identity was verified by size and anatomical location, regular pacemaker firing in cell-attached mode, and the presence of *I*_h_ current in response to a 50 mV hyperpolarizing step. D2-IPSCs were evoked using a platinum bipolar stimulator connected to a stimulus isolator (Warner Precision Instruments) placed in the slice caudal to the recorded cell bodies. All recordings are the average of 3–4 repetitions conducted once per minute with the 500 ms immediately preceding stimulation defined as the baseline. Recording and analysis of D2-IPSCs were conducted using AxoGraph software. All electrophysiological recordings and analyses were conducted blind to the genotype of the animal.

### Statistical analysis

**Figure 1.**
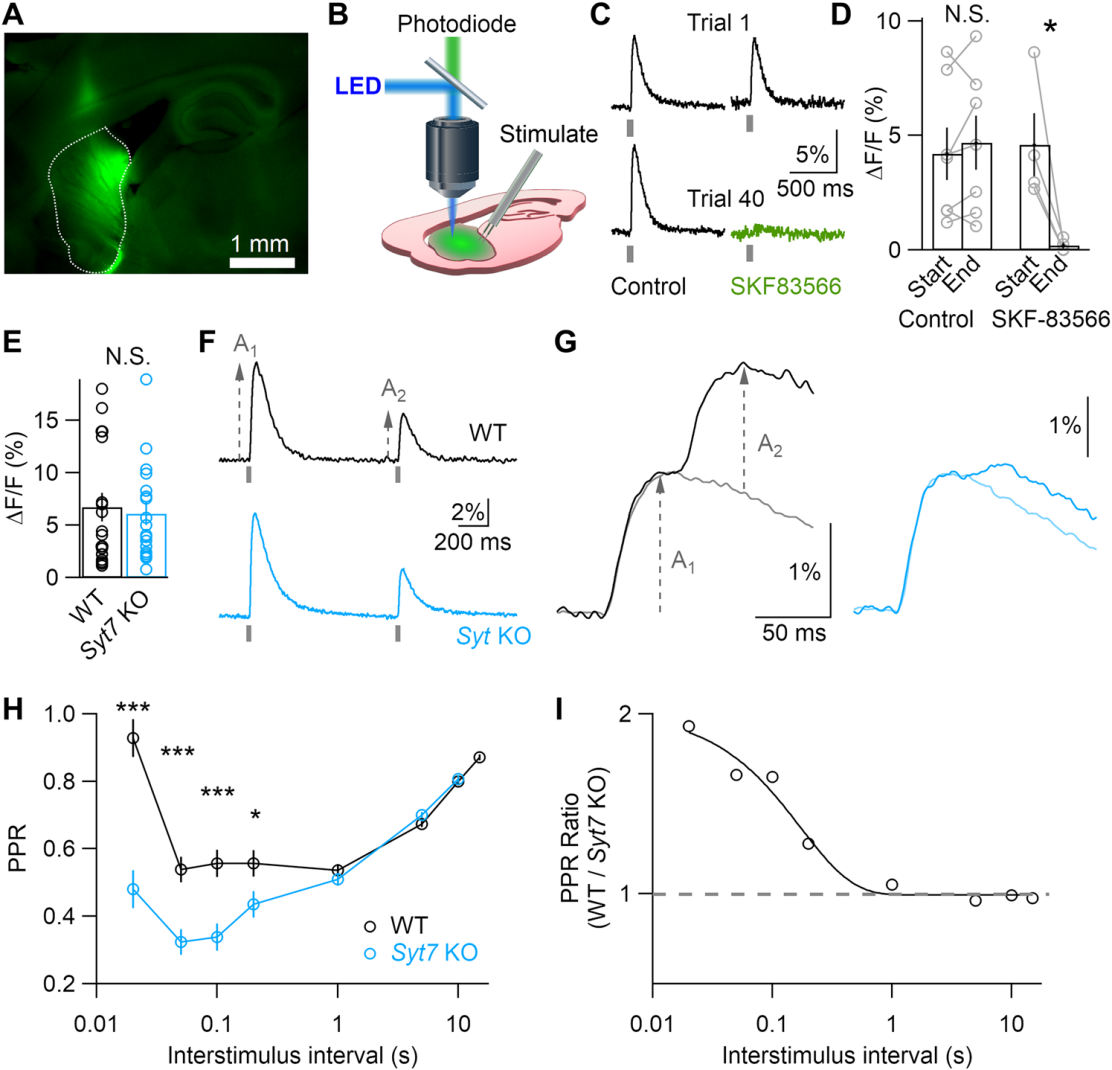
Syt7 reduces short-term depression of striatal dopamine release. ***A***, Representative fluorescent image of a sagittal brain slice with adeno-associated virus (AAV)-driven dLight expression in the dorsal striatum. ***B***, Experimental method used to capture fluorescence with an amplified photodiode detector mounted on an upright microscope. ***C***, Representative dLight transients recorded on the first and fortieth trials during an experiment with (right) or without (left) wash-in of the D1 antagonist SKF83566. ***D***, Average response amplitudes for slices kept in control ACSF or superfused with the D_1_R antagonist SKF83566. ***E***, Response amplitudes recorded from WT (*N* = 18) and *Syt7* KO animals (*N* = 18). ***F***, Representative responses to paired-pulse stimulation at 1 s intervals; each trace represents the average of five trials. ***G***, As in ***F***, but for 50 ms stimulus interval, illustrating how the response to a single stimulus (A_1_) was used to determine the amplitude of the second response (A_2_). ***H***, Paired-pulse ratios (PPR = A_2_/A_1_) at varying interstimulus intervals. ***I***, Ratio of WT and *Syt7* KO PPR values, showing single exponential fit with 170 ms decay. Data in all figures expressed as mean ± SEM. Statistical significances assessed by Kruskal–Wallis (***E***) or unpaired Student's *t* test (***D***,***H***) are shown as *p < 0.05 and ***p < 0.001.

**Figure 2.**
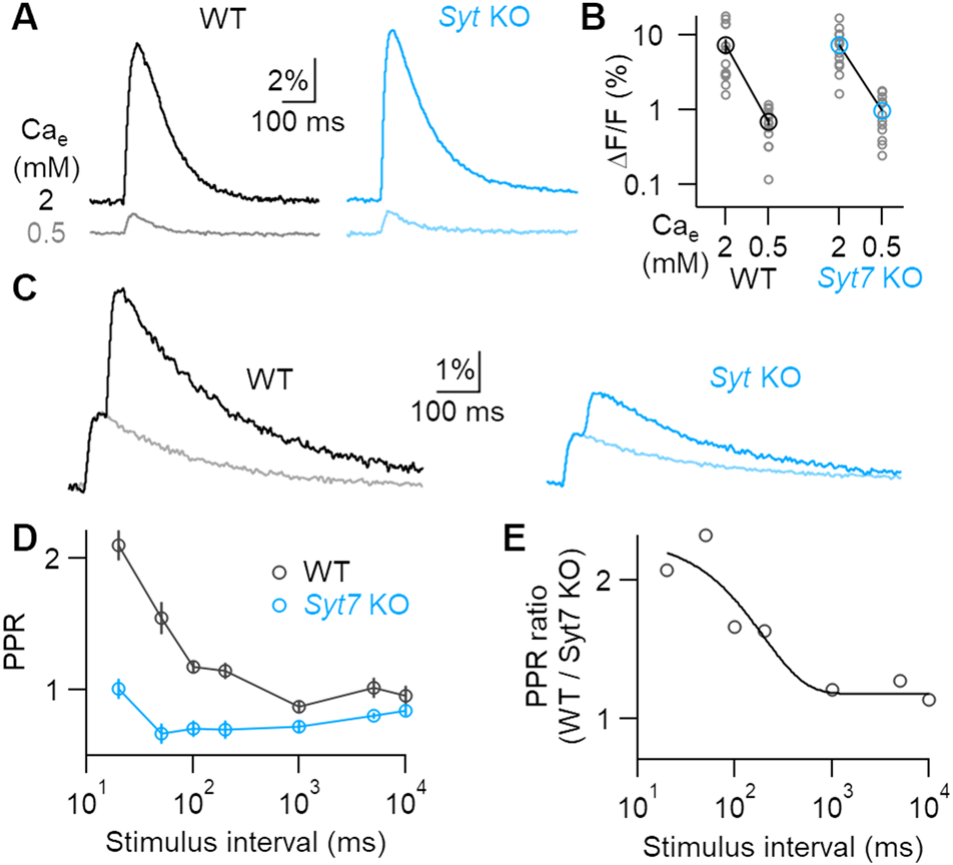
Lowering release probability reveals SYT7-mediated facilitation at striatal terminals. ***A***, Representative dLight transients in WT and *Syt7* KOs recorded from the same brain slice in high and low Ca_e_. ***B***, Response amplitudes recorded from slices where Ca_e_ was lowered from 2 to 0.5 mM (WT, *N* = 14; *Syt7* KO, *N* = 13). ***C***, Representative fluorescent transients evoked by paired-pulse stimulation at 50 ms intervals (dark traces) and single stimuli (light traces) in 0.5 mM Ca_e_. ***D***, Average paired-pulse ratios in 0.5 mM Ca_e_ at varying interstimulus intervals in WT (*N* = 15) and Syt7 KOs (*N* = 15). ***E***, Ratio of average WT and Syt7 KO PPR, showing a single exponential fit with 196 ms decay.

**Figure 3.**
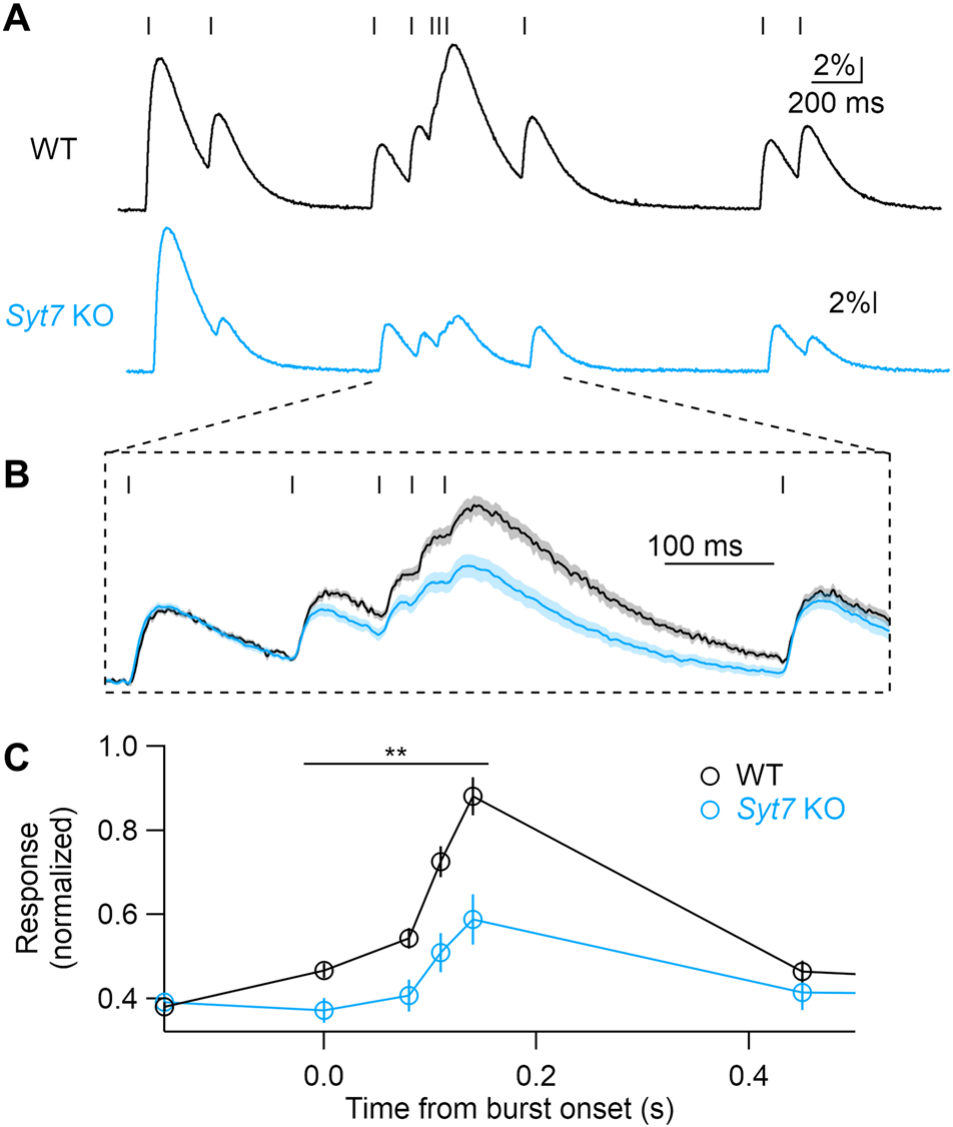
SYT7 promotes dopamine release during phasic bursts. ***A***, Stimulus train modeled on in vivo recordings from SNc dopamine neurons and representative dLight responses from WT and *Syt7* KO animals. ***B***, Averaged responses from WT (*N* = 9) and KO (*N* = 6), normalized to the initial response amplitude. ***C***, Average normalized peak amplitudes for each stimulus, relative to the onset of the phasic burst. Statistical significance assessed by Student's *t* test, **p < 0.01.

**Figure 4.**
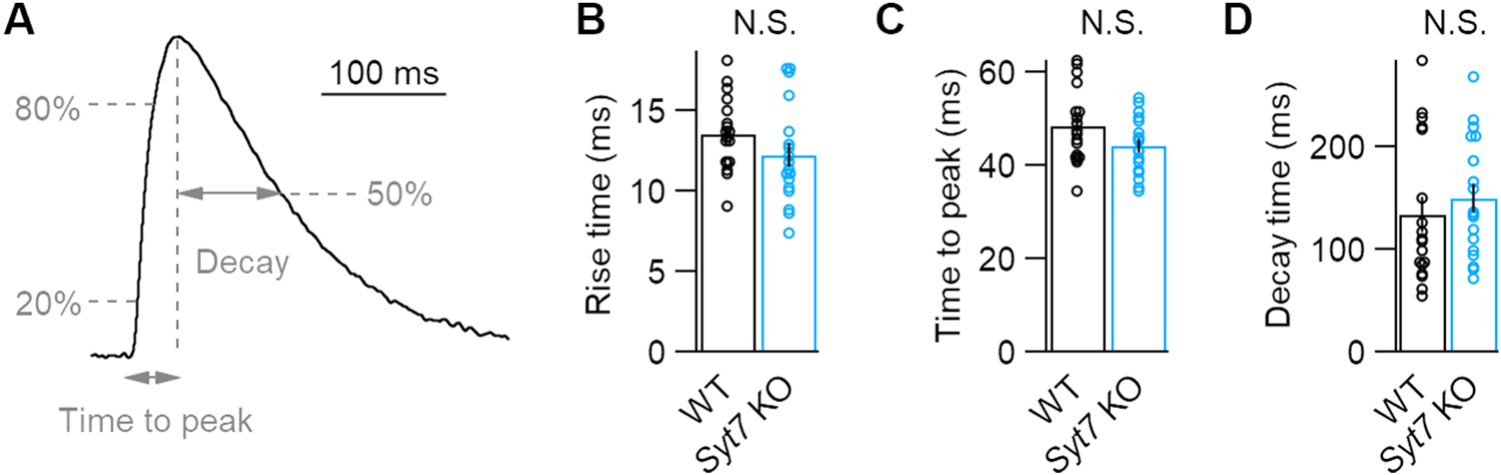
The kinetics of dLight responses is unaffected by Syt7 depletion. ***A***, Representative striatal dLight transients showing methods used to calculate kinetics. ***B–D***, Averaged kinetics of dLight transients for both genotypes, as measured by the 20–80% rise time (***B***), time to peak (***C***), and 50% decay time (***D***). *N* = 18 for both genotypes. No significant differences were determined by unpaired Student's *t* tests (***B***,***C***) or Kruskal–Wallis test (***D***).

**Figure 5.**
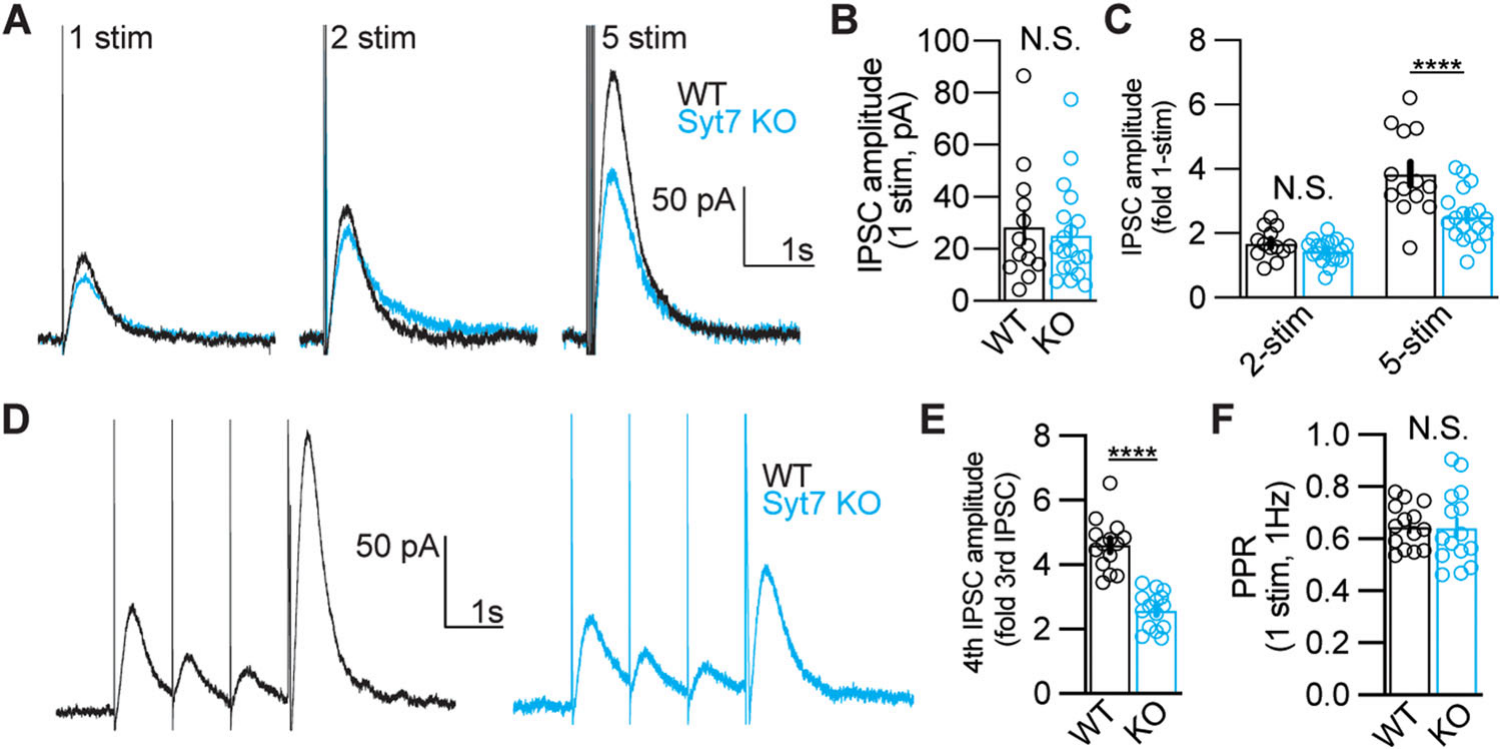
Syt7 mediates the facilitation of somatodendritic dopamine release. ***A***, Representative D2-IPSCs induced by a single electrical stimulus (1 stim), a pair of stimuli at 40 Hz (2 stim), or a train of five stimuli at 40 Hz (5 stim). ***B***, Amplitude of D2-IPSCs elicited by single stimuli in WT and *Syt7* KO animals (WT, 28.4 pA ± 6.2 pA, *N* = 13 cells from four animals); KO, 25.3 ± 4.2 pA (*N* = 19 cells from four animals); *p* = 0.79, Mann–Whitney *U* test). ***C***, Amplitude of D2-IPSCs induced by two or five stimuli at 40 Hz, normalized to the response elicited with one stimulus (2 stim—WT, 1.69 ± 0.13-fold 1 stim; KO, 1.45 ± 0.08-fold 1 stim, *p* = 0.64; 5 stim—WT, 3.84 ± 0.36-fold 1 stim, Syt7 KO, 2.52 ± 0.18-fold 1-stim, *p* < 0.0001; Sidak test following two-way ANOVA, same *N* as in ***A***). ***D***, Unmasking of facilitation protocol using three stimuli at 1 Hz, followed by a five-stimulus train at 100 Hz. The facilitated release is measured as the ratio of the five-stimulus IPSC to the third IPSC at 1 Hz. ***E***, Quantification of facilitation in WT and *Syt7* KO animals (WT, 4.61 ± 0.21, *N* = 14 cells, three animals; KO, 2.59 ± 0.15, *N* = 15 cells, three animals; *p* < 0.0001, unpaired *t* test). ***F***, PPR measured for the first two 1 Hz stimuli (WT, 0.65 ± 0.02; KO, 0.64 ± 0.04, *p* = 0.90 unpaired *t* test, same *N* as in ***E***). All data are presented as mean ± SEM with individual data points displayed. Statistical significances are shown as ****p < 0.0001.

Group sizes were not determined a priori to conducting experiments. All statistical analyses are indicated in figure legends for corresponding data sets. Statistical analyses were conducted in Igor Pro 9 (Figs. 1–4) or GraphPad Prism (Fig. 5). The normality of data was determined by the Shapiro–Wilk test. All data are presented as mean ± SEM.

## Results

To determine whether or not SYT7 impacts striatal dopamine signaling, we monitored evoked release in acute slices using the genetically encoded fluorescent dopamine sensor dLight1.3b (Patriarchi et al., 2018). Adeno-associated viral vector was injected into the dorsal striatum of WT and *Syt7* KO mice. Acute sagittal brain slices were prepared for imaging and electrophysiology after allowing at least 1 week for the virus to drive dLight expression (Fig. 1*A*). dLight was excited with dim light (∼1 mW/mm^2^), and fluorescence emission was captured by an amplified photodiode detector (Fig. 1*B*). To isolate action potential-driven dopamine release, axon terminals were activated by extracellular stimulation in the presence of antagonists for nicotinic, D2, and GABA receptors. Fluorescent transients showed fast kinetics suitable for investigating short-term plasticity, and minimal photobleaching over successive trials permitted prolonged imaging of the same region (Fig. 1*C,D*). Transients likely reflected local elevations in dopamine, as dLight binds dopamine with high specificity. dLight binds norepinephrine and epinephrine with 70- and 40-fold lower affinity than that of dopamine, respectively (Patriarchi et al., 2018), but these transmitters are released at much lower levels than dopamine in the dorsal striatum (Ihalainen et al., 1999). Moreover, fluorescent transients were reduced by 95 ± 3% (*N* = 4) by the selective D1 receptor antagonist (SKF83566, 10 μM), further suggesting that fluorescence was driven by dopamine binding to dLight (Fig. 1*C,D*).

Short-term plasticity is often quantified by stimulating twice in rapid succession and measuring the “paired-pulse ratio” (PPR) of synaptic responses. At low-release probability synapses, SYT7 can increase the amount of transmitter released by the second stimulus for intervals less than ∼200 ms, leading to paired-pulse ratios >1 (Jackman and Regehr, 2017). At high-release probability synapses that express SYT7, such as cerebellar Purkinje cell terminals, the initial stimulus depletes the pool of releasable vesicles and decreases the amplitude of the second response. Thus, although SYT7 does not produce overt facilitation at Purkinje cell terminals, it combats depression during the second stimulus (Turecek et al., 2017).

We measured paired-pulse ratios in WT and *Syt7* KO mice to assess the short-term plasticity of striatal dopamine release (Fig. 1*E*). For stimulus intervals of 1 s, dLight transients in WT and *Syt7* KO slices both exhibited ∼50% depression and recovered over the course of 10 s (Fig. 1*F*). However, a marked difference between genotypes appeared at higher stimulus frequencies (Fig. 1*G,H*). At the shortest stimulus interval of 20 ms, PPR approached 1 in WT animals (0.93 ± 0.06), while *Syt7* KOs exhibited pronounced depression (0.48 ± 0.04). To more clearly show the contribution of SYT7 to short-term plasticity, we calculated the ratio of WT and *Syt7* KO PPR values (Fig. 1*I*). This method offsets the similar depression seen in both genotypes at longer stimulus intervals. PPR was twofold higher in WTs than that in KOs at short intervals, and this difference decayed with an exponential time course of 170 ms, typical of a classical facilitation time course seen in low-release probability synapses (Jackman and Regehr, 2017). Thus, although dopamine terminals have a high initial release probability, SYT7 counteracts vesicle depletion to maintain response amplitudes during high-frequency stimulation.

Hidden facilitation can be revealed at synapses that exhibit net depression by decreasing external Ca^2+^ (Ca_e_), which reduces release probability and vesicle depletion. Lowering Ca_e_ from 2 to 0.5 mM reduced single-stimulus response amplitudes similarly in both genotypes (WT, 87 ± 2%; Syt7 KO 86 ± 2%; *p* = 0.68; Fig. 2*A,B*). This indicates that SYT7 does not significantly affect the calcium dependence of dopamine release, as has been shown at other synapses (Bacaj et al., 2013; Liu et al., 2014; Jackman et al., 2016). The calcium dependence of dLight responses was similar to previous measures of striatal dopamine release performed with fast-scan cyclic voltammetry (FSCV; Ford et al., 2010). However, lowering Ca_e_ revealed a qualitative difference in paired-pulse plasticity between genotypes. WT synapses showed twofold facilitation at short stimulus intervals, while *Syt7* KO synapses exhibited no facilitation or mild depression (Fig. 2*C,D*). The ratio of PPR values from WT and *Syt7* KOs produced a curve similar to that seen in high external Ca_e_, which decayed with a time course of 190 ms (Fig. 2*E*). Hidden forms of facilitation can also be revealed by applying high-frequency stimuli after predepressing synapses with low-frequency stimulation (Turecek et al., 2016; Doussau et al., 2017). This stimulation paradigm is highly relevant to dopamine neurons, whose in vivo firing patterns switch between tonic pacemaker activity and phasic bursts. Phasic bursts produce superlinear increases in striatal dopamine release (Schultz et al., 1997; Tsai et al., 2009). To explore how SYT7 affects release during physiological patterns of activity, we stimulated axons with spike train patterns previously recorded from SNc dopamine neurons in vivo (Schiemann et al., 2012). The spike pattern contained periods of tonic firing (instantaneous firing rates ranging from 1 to 6 Hz) as well as a phasic burst (12–33 Hz). Release during the first three tonic stimuli was similar in WT and *Syt7* KO slices (Fig. 3*A*). However, at the onset of a phasic burst, dLight fluorescence increased rapidly in WTs, often reaching the amplitude of the initial predepressed response. In contrast, release during the phasic burst in KOs was significantly depressed (Fig. 3*B,C*). These data show that SYT7 promotes release during phasic activity and suggest that short-term plasticity plays an important role in the nonlinear increase in striatal dopamine release seen in vivo when neurons fire bursts.

In addition to facilitation, SYT7 promotes asynchronous release (Chen et al., 2017), which could in principle prolong dopamine release and extend the time course of dLight transients (Diamond and Jahr, 1995). However, SYT7-driven asynchronous release does not significantly alter postsynaptic response kinetics at other synapses (Liu et al., 2014; Chen et al., 2017), and a recent study reported that genetically depleting SYT7 did not affect the kinetics of fluorescent transients produced by the optical glutamate sensor iGluSnFR (Wu et al., 2023). Nonetheless, we measured the time course of dLight1.3b transients induced by a single stimulus to determine whether SYT7 affects dopamine release kinetics. Although dLight1.3b can buffer dopamine at release sites and slightly prolong D2-IPSCs, fluorescent transients provide an approximate measure of synaptic dopamine concentrations due to dLight's low affinity and rapid on/off kinetics (Condon et al., 2021). We found that the rise time (20–80%), the time to the peak of the dLight transient, or the time it took for transients to decay to 50% of peak amplitude were all similar across genotypes (Fig. 4). Thus, SYT7 does not drive sufficient asynchronous release from striatal dopamine terminals to affect the kinetics of dLight transients, though these experiments likely lack the sensitivity required to detect subtle changes in the time course of release.

Our results thus far indicate that SYT7 drives short-term facilitation of release from striatal dopamine terminals during transitions from tonic and phasic firing. Dopamine is also released in the midbrain from somatodendritic compartments where it inhibits cell firing (Beckstead et al., 2004). Recent reports have described a role of SYT7 in somatodendritic dopamine release similar to the above-described role in terminal regions (Hikima et al., 2022). To confirm SYT7's role in modulating dopamine release from the somatodendritic compartment, we performed whole-cell voltage-clamp recordings of D2-IPSCs in the SNc from WT and *Syt7* KO slices. D2-IPSCs induced by a single electrical stimulus, or a pair of stimuli at 40 Hz, were unchanged in *Syt7* KOs (Fig. 5*A–C*). However, the amplitude of D2-IPSCs elicited by trains of five stimuli at 40 Hz was lower than that in KO animals (Fig. 5*C*). To examine whether SYT7-mediated facilitation could be revealed by predepressing synapses with low-frequency stimulation, we stimulated the SNc with three prepulses at 1 Hz, followed by a phasic train of five pulses at 100 Hz (Fig. 5*D*). Facilitation of release was measured as the ratio of the final prepulse IPSC (third) and the IPSC induced by the 100 Hz phasic stimulus train (fourth; Beckstead et al., 2007). In WT animals, the phasic IPSC was ∼4.6-fold larger than the predepressed IPSC, a greater ratio than that seen without a series of prepulses (Fig. 5*E*). However, in *Syt7* KO slices, the ratio of prepulse to phasic IPSC was significantly lower than that in WT slices, and did not differ from that seen without predepression in KO slices. Paired-pulse ratios for stimuli presented at 1 Hz were similar in WT and KO animals (Fig. 5*F*), consistent with SYT7's minimal contribution to release at low stimulation frequencies in other synapses (Jackman et al., 2016). Thus, Syt7-mediated facilitation of dopamine release also reduces depression of release during high-frequency stimulation at somatodendritic sites.

## Discussion

Here, we report that SYT7 mediates a form of hidden facilitation that limits short-term depression of dopamine release at both somatodendritic and terminal sites. SYT7 significantly increases dopamine release during phasic bursts, despite the fact that both axonal and dendritic synapses exhibit a high initial release probability and overt synaptic depression. A mixture of facilitation and depression of dopamine release was previously inferred from in vivo electrochemical recordings of striatal dopamine concentrations during midbrain stimulation (Montague et al., 2004). Similar hidden facilitation has been observed at nondopaminergic depressing synapses, where SYT7 maintains release during sustained firing (Chen et al., 2017; Turecek et al., 2017). Our experiments highlight the utility of dLight1.3b for investigating rapid dopaminergic signaling, which could be extended to assess how presynaptic short-term plasticity impacts dopamine release in vivo.

Despite progress in understanding the molecular basis of dopamine release, the Ca^2+^ sensors that regulate release in terminals and the somatodendritic compartment are not fully resolved. Dopamine release relies on some of the same machinery that supports fast, Ca^2+^-dependent neurotransmitter release from nondopamine synapses: Deletion of the active zone scaffolding protein RIM abolishes release in both terminals and dendrites (Liu et al., 2018; Robinson et al., 2019). Despite this shared necessity of RIM, dendrites and terminals may employ different Ca^2+^ sensors for release. The vesicular Ca^2+^ sensor SYT1 is required for release from terminals (Banerjee et al., 2020), but there are mixed results regarding its necessity for release from somatodendritic compartments (Delignat-Lavaud et al., 2021; Lebowitz et al., 2023). Variable results have also been observed for SYT7's role in the somatodendritic compartment. Intracellular application of a SYT7 antibody significantly reduced autaptic D2-IPSCs in response to stimulus trains (Hikima et al., 2022). However, dopamine release triggered by stimulus trains measured using FSCV was not different in *Syt7* KO mice compared with controls, though a double KO of *Syt7* and *Syt4*, a non–Ca^2+^-binding synaptotagmin isoform, displayed a marked reduction of release (Delignat-Lavaud et al., 2022). Notably, the release was also decreased in mice that were heterozygous for *Syt7* KO, suggesting an outsized role of SYT7 (Delignat-Lavaud et al., 2022). Our results suggest that *Syt7* KO alone is sufficient to reduce dopamine release triggered by stimulus trains in both somatodendritic and terminal compartments.

A potential limitation of this study is the use of global *Syt7* KO animals, which might lead to compensatory changes that confound the interpretation of our results. However, previous studies have found that global *Syt7* depletion selectively abolished presynaptic plasticity, without changing other neuronal or synaptic properties (Bacaj et al., 2013; Jackman et al., 2016). Moreover, facilitation is restored in global knockouts in a cell-autonomous manner by presynaptic rescue of *Syt7* (Jackman et al., 2016; Turecek et al., 2017). In biochemical assays, SYT7 remains bound to calcium for hundreds of milliseconds (Hui et al., 2005), similar to the time course of facilitation we observed for dopamine release. Hence, the most parsimonious interpretation of our results is that SYT7 binds to presynaptic calcium following an action potential, and SYT7 activation potentiates dopamine release for tens to hundreds of milliseconds. Like RIM, the conserved role of SYT7 in release from terminals and the somatodendritic compartment indicates mechanistic similarities in the sites that underlie evoked dopamine release at each site. The intrinsic modulation of dopamine release by short-term plasticity, and how this plasticity acts in concert with extrinsic regulators of release including acetylcholine, will remain critical avenues of investigation in understanding how the basal ganglia encodes the multitude of behaviors associated with dopamine signaling.

## Synthesis

Reviewing Editor: Arvind Kumar, KTH Royal Institute of Technology Decisions are customarily a result of the Reviewing Editor and the peer reviewers coming together and discussing their recommendations until a consensus is reached. When revisions are invited, a fact-based synthesis statement explaining their decision and outlining what is needed to prepare a revision will be listed below. The following reviewer(s) agreed to reveal their identity: Christopher Ford.

Synthesis

Here authors have identified how dopamine neurons which fire in tonic and bursting modes can release dopamine during bursts. As dopamine is often thought to have a high probability of release, it has not been clear how dopamine neurons can subsequently release dopamine during bursts and why this release facilitates. While past work has identified SYT1 as the Ca2+ sensor for high Pr release, the sensor that drives facilitation has remained unclear. This work identifies that sensor.

Both reviewers think that authors have addressed an interesting and relevant problem about dopamine release. However, the reviewers think that we need some additional control in addition to clarification on methods.

Reviewer #1

Here the authors examine the role of SYT7 in regulating axonal and somatodendritic dopamine release by comparing control to Syt7 KO animals. The authors use the combination of rapid dLight photometry measurements in the striatum together with somatodendritic electrophysiology recordings of D2-R mediated synaptic currents in SNc neurons. Using these approaches they find that SYT7 plays a prominent role in regulating short term facilitation of dopamine release. Through a nice set of experiments where they lower extracellular calcium and examine bursts of stimuli following low frequency stimulation they find that SYT7 is an important Ca2+ sensor that limits short-term depression of dopamine release which allows for facilitation during bursts of activity. This combined with previous work defining the role of SYT1 in dopamine neurons makes an important contribution to the field.

The experiments are well-performed, the conclusions appropriately drawn from the data and the manuscript is well written. In my opinion the manuscript is suitable for publication in the present form, and no further experiments or additions to the text are needed.

Reviewer #2

This study investigated the role SYT7 plays in regulating dopamine release. By integrating dopamine imaging using a genetically encoded sensor light and with electrophysiological methods (D2-IPSCs), this study uncovers the role of SYT7 in enhancing dopamine release, particularly during the transition from tonic to phasic firing. Overall, this study design is straightforward, and the conclusion is justified. I only have a few minor comments:

1. Methodologically, doing in vitro dLight imaging in striatal slices is very useful. I would suggest including a control experiment using a control sensor for dLight (or blockers) to show the specificity of this approach. If another method, e.g., using D2-IPSCs, could be used to confirm the involvement of Syt7 in the striatal release of DA, that would be great.

2. The comparison of PRR between the WT and SYT7 KO is decent and provides good evidence for the findings of this paper. It is unclear for each of the measurements (per slice) whether only one trial was done at a given interval or several trials, then taking the average. A little bit of clarification in the method section would be helpful. Perhaps it would be beneficial to demonstrate the repeatability of this calculation and its resilience against variations in sensor expression and response levels.

3. Referring to the possible involvement of Syt7 in asynchronous release, the author analyzed the single-pulse induced dLight1.3b imaging. I wasn't so convinced that this experiment could address the hypothesis.

Minor:

1. The figure numbering in this paragraph appears incorrect (near lines 202-203). Should be "Fig. 2"?

2. "Adult mice (P30-P90) were anaesthetized with ketamine/xylazine (100/10 mg/kg), placed in a stereotaxic apparatus (Kopf), and supplemented with 2% isoflurane." In my experience, the animals would not be able to survive 2% + Ketamine/Xylazine for a long time during the surgery. Please make sure the description is accurate in your experiment.

## Author Response

We thank the reviewers for carefully assessing our manuscript, and we were very pleased that both reviewers shared positive views of our work. We endeavored to address all the issues raised by reviewer #2, and hope that these changes improved the manuscript. All edited sections have been colored in blue. We also altered the color scheme for genotypes in the figures on the advice of colorblind colleagues.

Reviewer #1 Here the authors examine the role of SYT7 in regulating axonal and somatodendritic dopamine release by comparing control to Syt7 KO animals. The authors use the combination of rapid dLight photometry measurements in the striatum together with somatodendritic electrophysiology recordings of D2-R mediated synaptic currents in SNc neurons. Using these approaches they find that SYT7 plays a prominent role in regulating short term facilitation of dopamine release. Through a nice set of experiments where they lower extracellular calcium and examine bursts of stimuli following low frequency stimulation they find that SYT7 is an important Ca2+ sensor that limits short-term depression of dopamine release which allows for facilitation during bursts of activity. This combined with previous work defining the role of SYT1 in dopamine neurons makes an important contribution to the field.

The experiments are well-performed, the conclusions appropriately drawn from the data and the manuscript is well written. In my opinion the manuscript is suitable for publication in the present form, and no further experiments or additions to the text are needed.

We thank the reviewer for their positive assessment and feedback of our work.

Reviewer #2 This study investigated the role SYT7 plays in regulating dopamine release. By integrating dopamine imaging using a genetically encoded sensor light and with electrophysiological methods (D2-IPSCs), this study uncovers the role of SYT7 in enhancing dopamine release, particularly during the transition from tonic to phasic firing. Overall, this study design is straightforward, and the conclusion is justified. I only have a few minor comments:

We thank the reviewer for their positive assessment of our work and suggestions to enhance the data and text. We have addressed each comment and point-by-point responses are below.

1. Methodologically, doing in vitro dLight imaging in striatal slices is very useful. I would suggest including a control experiment using a control sensor for dLight (or blockers) to show the specificity of this approach. If another method, e.g., using D2-IPSCs, could be used to confirm the involvement of Syt7 in the striatal release of DA, that would be great.

We have added additional control data (Figure 1C,D; lines 175-177) showing the loss of fluorescent transients when slices were superfused with the selective D1R agonist SKF-83566. We also added additional text describing the specificity of dLight for dopamine. In their original description of dLight1.3b, Patriarchi et al (2018) showed that dLight does not respond to a wide array of transmitters, and although it does bind to norepinephrine and epinephrine, it does so with an affinity orders of magnitude lower than for dopamine. We now state this in the text, along with the fact that norepinephrine is released at much lower levels than dopamine in the dorsal striatum where recordings were made (lines 172-175).

2. The comparison of PRR between the WT and SYT7 KO is decent and provides good evidence for the findings of this paper. It is unclear for each of the measurements (per slice) whether only one trial was done at a given interval or several trials, then taking the average. A little bit of clarification in the method section would be helpful. Perhaps it would be beneficial to demonstrate the repeatability of this calculation and its resilience against variations in sensor expression and response levels.

We thank the reviewer for pointing out this ambiguity. Each interstimulus interval was presented over multiple (4-10) trials and the responses averaged to generate the final data point. We have added additional text to the methods (lines 115-120) to explain these experiments more clearly. We also used linear regression to test whether paired-pulse ratios were affected by response amplitudes, and found no relationship (lines 120-121).

3. Referring to the possible involvement of Syt7 in asynchronous release, the author analyzed the single-pulse induced dLight1.3b imaging. I wasn't so convinced that this experiment could address the hypothesis.

We agree with the reviewer that our experiments analyzing the time-course of transients are likely not sensitive enough to directly observe contributions of asynchronous release. However, we have kept the time-course data in the manuscript to serve as a basis of comparison for future experiments that examine dLight transients at high speeds in the striatum. We have altered the results section and added additional text (lines 234-247) to describe the rationale for these measurements and explicitly acknowledge the limitation of interpretations on asynchronous release.

Minor:

1. The figure numbering in this paragraph appears incorrect (near lines 202-203). Should be "Fig. 2"? We thank the reviewer for pointing out this error and have corrected it in the text.

2. "Adult mice (P30-P90) were anaesthetized with ketamine/xylazine (100/10 mg/kg), placed in a stereotaxic apparatus (Kopf), and supplemented with 2% isoflurane." In my experience, the animals would not be able to survive 2% + Ketamine/Xylazine for a long time during the surgery. Please make sure the description is accurate in your experiment.

We have performed hundreds of intracranial injections using this combination of anesthetics. In our experience this protocol maintains anesthesia throughout surgery, does not result in death, and animals recover quickly after surgery. However, our description was slightly inaccurate: the concentration of isoflurane used was adjusted between 1-2% and animals were monitored to ensure steady respiration, and we have updated the methods section to reflect this (line 88).
